# Gunshot Wound in the Abdomen—Sloughing—Blood Evacuated from Bowels

**Published:** 1849-06

**Authors:** James Bryan

**Affiliations:** Philadelphia. Professor of Surgery in Geneva Medical College, N. York


*Gunshot Wound in the Abdomen—Sloughing—Blood evacuated from Bowels. Cured.* By James Bryan, M. D., of Philadelphia. Professor of Surgery in Geneva Medical College, N. York.

Sept. 9th, 1848, I was called in consultation with Dr. Gardiner, of Francisville, to see a young man named George Merrine, aged about twenty-five years. On examination, we found that a surface about four inches square, one inch and a half below the umbilicus, was torn up, and *infiltrated* with seed-bird shot, powder, gun-wadding, and portions of the patient's shirt and pantaloons. The surface was as black as charcoal, and torn up into rough elevations and depressions; and blood ran freely from the surface. The pulse was slow and weak, and the patient very faint, with considerable nervous tremors. Any attempt to remove the pieces of cloth, wadding, or shot, was followed by profuse hemorrhage. Twelve or fifteen of the loose shot were removed, and the patient directed to take forty drops of laudanum; a large flax-seed poultice to be applied to the abdomen, and perfect rest. The laudanum to be repeated during the night, if the restlessness was not controlled.

10th. Reaction has taken place—the pulse strong and full—considerable pain in the abdomen. We directed thirty leeches to be applied around, and over the wound. The poultice brought away a number of shot, and pieces of cloth and wadding. In the evening he was bled from the arm fifteen ounces, and the laudanum repeated. Diet, gruel and cold water. A blue pill was given with the laudanum.

11th. Symptoms about the same—pain continues in the abdomen—pulse about eighty. The bowels having been freely evacuated before we saw him, no attempt was made, to-day, to move them. Continue poultice; to be renewed two or three times during the day and night; forty drops of laudanum in the evening. The pulse having risen again in the afternoon, the patient was freely bled from the arm. Some twenty shot were removed from the wound, and the hemorrhage from the points of lodgment, was not great.

12th. Clots of blood have been discharged per ano, during the night mixed with pus, accompanied with considerable pain. The patient begins to desire food; chicken-water in small quantities was directed, with a pill of one grain of opium, with one and a half grains of acetate of lead, to be taken every four hours.

Afternoon. The surface of the wound is beginning to throw off a slough. Fifteen or twenty shot were removed, with additional portions of powder and wadding. Two copious discharges of blood, per ano, have taken place since morning. Great debility, and severe griping pains in the bowels. Continue the opium and acetate of lead pill, every two hours, with a starch and morphia clyster. Renew the poultice.

13th. Feels comfortable—the skin moist—the pulse soft and regular; about seventy-five per minute—has had two light stools since the clyster last evening—less pain on pressure, and little tenderness—appears in a condition which may be termed *half opiated.* The blood was not very carefully examined, for what we feel little doubt it contained, viz., shot. The probe, from the beginning, would easily enter many of the shot holes, some four or five inches. Continue the pills every four hours—also chicken or beef teas, every hour.

14th. Continues to improve. The slough may be scraped off with the finger nails, bringing with it powder, shot and pieces of cloth. Patient asks for food. Bowels moved but once during the night, and the stool but slightly colored with blood. Diminish the pills to two, to-day—renew the poultice—diet as before. This treatment continued, with a diminution of the opium, to the

17th, when the slough was nearly off, and about fifty shot had been removed from the wound. A large ulcer remained, which it was dangerous to meddle with, on account of its proximity to the bowels. The fluid diet was continued a few days longer—the ulcer dressed with mercurial ointment, and occasionally touched with nitrate of silver. The patient got well without a bad symptom. The ulcer remained for some four months, before it entirely healed; but, at the present writing, it is well, and the patient at work at his trade, that of a cooper.

The young man had been out gunning, for seed birds, and having loaded his gun, sat down on a heap of logs or rails. The barrel of the gun was in his hands, and allowing it to slide too fast through them, the butt struck the ground, when the muzzle was within two inches of his abdomen. The jar made the gun, which was half cocked, go off, and the whole load was received in his body.

May 10th, 1849.

